# Supplementary material for: Profiling of RNA 8-oxoG marks in Escherichia coli identifies critical intrinsic characteristics that contribute to 8-oxoG accumulation in bacteria
Source: RNA Biol. 2026 Nov 6;23(1):1–25. doi: 10.1080/15476286.2026.2753975 (PMC13761385; doi:10.1080/15476286.2026.2753975)
Supplement: Supplementary file 2 [file KRNB_A_2753975_SM7936.docx]

Table of Contents for Supplemental Tables for:

**Profiling of RNA 8-oxoG marks in *Escherichia coli* identifies critical intrinsic characteristics that contribute to 8-oxoG accumulation in bacteria**

Matthew R. Burroughs^1†^, Blanca I. Quiñones-Diaz^1^, Kobe B. Grismore^1^, Lydia M. Contreras^1,2,#^

^1^McKetta Department of Chemical Engineering, UT Austin, Austin, TX 78712

^2^Department of Molecular Biosciences, UT Austin, Austin, TX 78712

^#^Address correspondence to Dr. Lydia Contreras, [lcontrer@che.utexas.edu](mailto:lcontrer@che.utexas.edu)

**Supplemental Table Titles:**

***Table S1.*** DE-Seq2 output comparing input fraction of H2O2 vs CTRL exposed samples from 8-oxoG-RIP-Seq sequencing experiment in *E. coli*

**Table S2.** DE-Seq2 output comparing IP fraction vs input fraction of Untreated samples from 8-oxoG-RIP-Seq sequencing experiment in *E. coli*

***Table S3.*** DE-Seq2 output comparing IP fraction vs input fraction of H2O2 Treated samples from 8-oxoG-RIP-Seq sequencing experiment in *E. coli*

***Table S4.*** ShinyGO output of GO Biological Process pathways elevated in oxidation enriched RNAs derived from Untreated condition of 8-oxoG-RIP-Seq sequencing experiment in *E. coli*

***Table S5.*** ShinyGO output of GO Biological Process pathways elevated in oxidation depleted RNAs derived from Untreated condition of 8-oxoG-RIP-Seq sequencing experiment in *E. coli*

***Table S6.*** ShinyGO output of GO Biological Process pathways elevated in oxidation enriched RNAs derived from H2O2 Treated condition of 8-oxoG-RIP-Seq sequencing experiment in *E. coli*

***Table S7.*** ShinyGO output of GO Biological Process pathways elevated in oxidation depleted RNAs derived from H2O2 Treated condition of 8-oxoG-RIP-Seq sequencing experiment in *E. coli*

***Table S8.*** exomePeak2 output comparing pulldown fraction vs input fraction of Untreated samples from ChLoRox-Seq sequencing experiment

***Table S9.*** exomePeak2 output comparing pulldown fraction vs input fraction of H2O2 Treated samples from ChLoRox-Seq sequencing experiment

***Table S10.*** Intrinsic RNA metadata and multiple linear regression modeling predictions for *E. coli*

***Table S11.*** Intrinsic RNA metadata and multiple linear regression modeling predictions for *D. rad*

***Table S12.*** Intrinsic RNA metadata and multiple linear regression modeling predictions for *M. smeg*

***Table S13.*** Intrinsic RNA metadata and multiple linear regression modeling predictions for *S. one*

***Table S14.*** Intrinsic RNA metadata and multiple linear regression modeling predictions for *Ca. P. ubique*

***Table S15.*** ShinyGO output of GO Biological Process pathways elevated in model predicted oxidation enriched RNAs in *E. coli*

***Table S16.*** ShinyGO output of GO Biological Process pathways elevated in model predicted oxidation enriched RNAs in *M. smeg*

***Table S17.*** ShinyGO output of GO Biological Process pathways elevated in model predicted oxidation enriched RNAs in *S. one*

***Table S18.*** ShinyGO output of GO Biological Process pathways elevated in model predicted oxidation enriched RNAs in *Ca. P. ubique*

***Table S19.*** ShinyGO output of GO Biological Process pathways elevated in model predicted oxidation depleted RNAs in *E. coli*

***Table S20.*** ShinyGO output of GO Biological Process pathways elevated in model predicted oxidation depleted RNAs in *M. smeg*

***Table S21.*** SRA run information for each bacterial species analyzed using BV-BRC RNA sequencing analysis module

***Table S22.*** DE-Seq2 output comparing IP fraction vs input fraction of samples from 8-oxoG-RIP-Seq sequencing experiment in *D. rad*
